# Effects of Cashew Nut (*Anacardium occidentale* L.) Seed Flour in Moderately Malnourished Children: Randomized Clinical Trial

**DOI:** 10.1155/2020/6980754

**Published:** 2020-05-04

**Authors:** Ana Cristina Pereira de Jesus Costa, Mércia Kelly dos Santos Silva, Samae Batista de Oliveira, Luana Leite Silva, Alessandra Cruz Silva, Raidanes Barros Barroso, José de Ribamar Macedo Costa, Virlane Kelly Lima Hunaldo, Marcelino Santos Neto, Lívia Maia Pascoal, Márcia Caroline Nascimento Sá Ewerton Martins, Floriacy Stabnow Santos, Leonardo Hunaldo dos Santos, Gledson Weslley Pereira Santos, Maria Aparecida Alves de Oliveira Serra, Ariadne Siqueira de Araújo Gordon, Thiago Moura de Araújo, Márcio Flávio Moura de Araújo

**Affiliations:** ^1^Nursing Department, Maranhão Federal University, University Avenue, S/N, Imperatriz, MA, Brazil; ^2^Food Engineering Department, Maranhão Federal University, University Avenue, S/N, Imperatriz, MA, Brazil; ^3^Natural Sciences Department, Maranhão Federal University, University Avenue, S/N, Imperatriz, MA, Brazil; ^4^Physical Education Department, Maranhão Federal University, University Avenue, S/N, Imperatriz, MA, Brazil; ^5^Health Sciences Institute, University for International Integration of the Afro-Brazilian Lusophony, José Franco de Oliveira Street, S/N, Redenção, CE, Brazil

## Abstract

The monitoring and combined use of dietary supplements to restore adequate growth are paramount and highly recommended in child malnutrition, an important public health problem. The objective of this study was to analyze the effects of cashew nut seed flour in children with moderate malnutrition, treated at primary healthcare services. This is a randomized clinical trial conducted from April to October 2017 in the city of Imperatriz, Brazil. The sample comprised 30 children born at term, aged between 2 and 5 years, and newly diagnosed with malnutrition (60 days or less), randomized into experimental and control groups. The intervention consisted of daily intake of cashew nut seed flour. There was intragroup statistically significant difference in the glucose levels of children who were assigned to the control group (*p*=0.02) and in the glycated hemoglobin in the experimental group (*p* < 0.01). Intergroup analysis of glycated hemoglobin levels showed statistically significant differences in favor of the experimental group (*p*=0.01). HDL and LDL had, respectively, increased and decreased in the experimental group. The use of cashew nut seed flour in a 24-week period had positive effects on glycated hemoglobin, HDL, and LDL parameters in moderately malnourished children.

## 1. Introduction

Malnutrition continues to be a major public health problem, and about 150 million children under five years in developing countries are underweight. This results in disastrous implications of growth, development, and child survival [1, 2]. Malnutrition is mainly caused by lack of nutrients for the body metabolic activities, which can progress to complex manifestations over time [3].

The health status can be affected by the intake, absorption, transport, use, disposal, and reservation of nutrients resulting in nutritional imbalance, which can affect the entire body depending on the length [4]. According to the intensity and permanence, these changes will cause a greater or lesser degree of clinical signs and symptoms [5].

Regardless of the initial cause, nutritional imbalances can lead to changes in the body's reserves, resulting in biochemical/metabolic disorders [6]. Functional disorders are clinical manifestations detectable in the physical examination of severely malnourished individuals with widely recognized anatomical changes that may be reversible or evolve into loss of function or even death [2].

Child malnutrition is a risk factor for morbidity and mortality, and it is often associated with changes in the child health indicators [7]. The most used parameter in the analysis of the nutritional status is the assessment of growth through regular monitoring of standards such as weight‐for‐age, height-for-age, and weight-for-height. However, it is strongly recommended that other indicators be monitored, which makes it possible to expand knowledge of organic imbalances caused by malnutrition [8, 9].

Currently, there is no consensus on which dietary supplements are the best to treat malnutrition. Throughout the human history, supplements have been used in the treatment of diseases as part of popular culture, which is often discredited [10, 11]. However, given the scale of malnutrition and socioeconomic and health costs associated with it, there is growing scientific interest in this issue, given the fact that many families use and continue to use dietary natural products to combat malnutrition [3, 12].

Studies show that dietary supplementation may be effective in the recovery of malnourished children, when it is composed of essential nutrients for growth/development and when it is associated with periodic anthropometric, biochemical, and nutritional assessments [2, 13, 14].

The nutritional status of a population is directly related to socioeconomic factors, dietary patterns, and frequency of food intake. Thus, the biochemical evaluation of the use of food supplements is essential to prevent, diagnose, and treat malnutrition [15]. There is evidence of significant decrease in iron concentration, glucose, and serum albumin of malnourished children compared to healthy children [16].

Cashew nut (*Anacardium occidentale* L.) is a major Brazilian cash crop. It is a high value edible nut and a source of carbohydrates, proteins, phosphorous, iron, zinc, magnesium, fibers, and fatty acids [17]. However, the biochemical effects of the use of dietary supplements in malnourished children, such as the cashew nut seed flour, are not well established.

Blood biochemical parameters are rarely used in the evaluation of malnourished children in primary care services [18]. The inclusion of these data in the prevention and control of malnutrition contributes to the assessment of risk factors, inflammatory responses, fluid balance, and nutritional monitoring [19].

Biochemical measurements are used as a supplement for the nutritional, anthropometrical, and clinical assessments [18]. The evaluation of glycemia, total cholesterol and its fractions, minerals, and vitamins is also underutilized in the professional clinical routine [20].

Some clinical and metabolic complications of malnutrition can be detected based on these biochemical parameters, which often are related to infections, anemia, hypovitaminosis, and functional deficiencies. Such complications generate decreased physical capacity, stunted growth, and deficits in cognitive and behavioral development in children [21, 22]. The aim of this study was to determine whether the intake of cashew nut seed flour modifies the biochemical parameters of moderately malnourished children in Brazilian primary care services.

Some guidelines specify the treatment or management of moderate malnutrition, considering the impact of this problem on death from common diseases or increased risk for acute and/or severe malnutrition and its associated complications [23].

To the best of our knowledge, this is the first randomized clinical trial that used biochemical parameters to evaluate moderately malnourished children. We hypothesized that children with normal height and moderate malnutrition (when the weight deficit considered normal for the age is below 25 and 40%), aged from 2 to 5 years, showed improvement in biochemical parameters after the use of cashew nut flour.

## 2. Methods

### 2.1. Design

This is a randomized, open, prospective trial that was conducted from April 2017 to October 2017 in the city of Imperatriz, Brazil.

### 2.2. Participants

The study population was composed of malnourished or low weight children, according to the criteria of the World Health Organization (WHO), *z* score (−2<*z* < −3), and the indicator weight *x* height *x* body mass index (BMI) for age [3]. The sample comprised children registered with and assiduous in primary healthcare services of the study location.

The eligibility criteria were as follows: being a child born at term, age between 2 and 5 years, of both sexes, with a recent diagnosis of moderate malnutrition or low weight (60 days or less) recorded in the clinical record; absence of liver or kidney problems (based on medical records); and absence of allergy to products derived from cashew nuts. We excluded children with severe malnutrition (*z* < -3 for any anthropometric indices) and children receiving interventions for malnutrition.

The sample size was determined using a model for paired comparison data [24]. We have adopted the following parameters: nP: number of pairs; Z*α*/2: *α* error value of 1.96 (5%); Z*β*: ß error value of 0.84 (20%); SD: standard deviation of the difference between pairs, 0.20; and $\bar{D\;}$: mean difference between pairs: 0.15.

**Figure 1:**
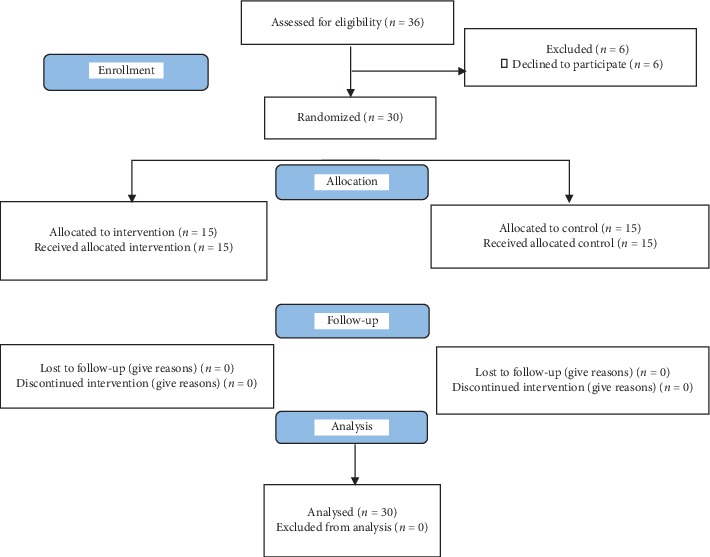
CONSORT flow diagram.

To obtain the sample size using the parameters mentioned above, we used results from a study reporting an assessment of nutritional status of Brazilian children [25], totaling approximately 14 pairs. Thus, the final sample was composed of 15 pairs (30 participants) distributed between the experimental and control groups, due to the difficulty of adherence of the participants who initiated the research, as the study flowchart shows in Figure 1:

### 2.3. Randomization

The randomization obeyed a rule for peer pairing using the following parameters: glycated hemoglobin, triglycerides, total cholesterol, and its fractions, with the objective to pair children with as similar values as possible for these parameters. After the collection of biochemical data, each child from each pair was randomized to case or control group by tossing a coin.

### 2.4. Cashew Nut Seed Flour Production

The cashew nut flour used in this study was obtained using roasted cashew kernels (*Anacardium occidentale* L.) collected at the same season, from an industry located in Fortaleza, Brazil. To obtain the flour, broken kernels were vacuum-packed and transported to the city of Imperatriz, where they were processed in a domestic blender for one minute. The flour was produced in a public laboratory of food and herbs. To ensure quality, the flour was submitted to tests in the laboratory according to the guidelines from the Adolfo Lutz Institute [26]. The results were as follows: water content was 4.8%, lipids were 43.2%, protein was 14.6%, fiber was 1.2%, ash was 2.6%, and carbohydrates were 33.6%. The percentage of carbohydrates was determined by difference. Approximately 388 kcal (kilocalories) was found in 100 grams of the cashew flour.

Micro and macroscopic analysis were made to ensure that the product had appropriate color, odor, flavor, and texture and to guarantee the absence of parasites fragments, insects, rodents, and dirt. The microbiological evaluation was carried out as required by the Resolution No. 12 of January 2, 2003, and Normative Instruction No. 01 of January 7, 2000 [27], in triplicate, using the most probable number per gram (MPN g-1) test of coliforms, the total coliforms at 45°C (thermotolerant) method, and the number of colonies forming units (CFU) of yeast and molds estimation [27]. No coliforms were found at a temperature of 35°C, indicating that all samples were in agreement with the Resolution No. 12 [28] and the current legislation. For yeasts and molds, all samples evaluated in the study were in the range 102–103, which is also in accordance with the current legislation, thus indicating that the process to obtain the flour was performed in a correct and efficient manner, ensuring the appropriate sanitary hygienic conditions of the final product. This analysis was guided by quality inspection guidelines of the Brazilian Association of Technical Standards (ABNT) No. 5426.

### 2.5. Intervention

Fifteen participants received the cashew nut flour in the ratio of two daily tablespoons (12 g), fractionated in the usual infant feeding (breakfast, lunch, and dinner), daily for a 24-week period. This amount is in accordance with scientific articles about food supplementation and undernutrition [29–31] and it is supported by food professionals consulted for this study (a food engineer and a nutritionist). All selected participants and children with adhesion superior to 75% completed the trial, with a total sample of 30 participants: 15 in the experimental group and 15 in the control group. Subsequently, we made home visits to deliver the remaining pouches (for the following four weeks). On these occasions, the used sachets were taken back for the records. Those participants with adhesion lower than 75% were excluded (discontinuity criteria). Participants from the control group received care as usual at the study locations.

Children's parents were asked to give up other offers of additional dietary supplements during the study period. Biochemical parameters were measured at baseline and at the end of the study. The laboratory analysis was made using 12-hour fasting blood samples, obtained at the beginning and at the end of the study. Analyses included fasting plasma glucose (FPG), glycated hemoglobin (HbA1c), triglycerides (TG), total cholesterol (TC), HDL-C, and LDL-C.

The blood samples were collected by a nurse technician who is a research collaborator. This person is qualified in the practice of handling blood samples and transportation of the samples for the laboratory. The samples were collected using the BD Vacutainer® vacuum collection system, and the venipuncture was performed in the antecubital fossa.

In addition, the children's parents answered questions about sociodemographic and economic characteristics of the family in the beginning of the study.

The FPG, TG, TC, HDL-C, and LDL-C analyses were made using the enzymatic assay method (Biosystems Spectrophotometer A15). The HbA1c analysis was measured using liquid chromatography (HPLC) (National Glycohemoglobin Standardization Program, United States).

Initially, we conducted descriptive analyses of sociodemographic, economic, and perinatal variables. Then, statistical tests were performed to verify possible changes in biochemical parameters within and between the groups, after 24 weeks of the intervention.

Throughout the process of data collection and intervention monitoring, nursing consultations were carried out with children from both groups and their parents. The results from the blood tests made before and after the intervention were disclosed for the parents.

### 2.6. Statistical Analysis

First, we tested normality of data using the Shapiro–Wilk test and the homogeneity of variance using the Bartlett's homogeneity test. If any of these tests were significant, then we used nonparametric tests. Except for weight and BMI, all variables presented nonparametric distribution. We used the paired Wilcoxon's *t*-test for intragroup analysis and the Wilcoxon–Mann–Whitney test for intergroup comparison [32]. The data were entered in a spreadsheet (Microsoft Excel 2016®) and analyzed using Statistical Analysis System (SAS)® at 5% level of significance [33].

### 2.7. Ethical Aspects

The study was registered in the Brazilian Ministry of Health's Brazilian Registry of Clinical Trials (Registro Brasileiro de Ensaios Clínicos, ReBec) under the number U1111.1213.9219. The study was approved by the Ethics Committee of the Maranhão Federal University (protocol no. 1,627,934) and all parents gave their oral and written informed consent to participate.

Children control group have received healthcare provided by nurses and pediatricians from the local health service. That is, they had access to child growth and development consultations and guidance on healthy nutrition.

## 3. Results

A predominance of females was observed in both groups: control (53.33%) and experimental (73.33%). The groups were also similar in mean age: experimental group (2.93 years) and control group (2.46 years).

After the intervention, there was intragroup statistically significant difference (*p*=0.02) regarding FPG in the control group and regarding HbA1c in the experimental group (*p* < 0.01). With regard to the postintervention serum lipids dosage, we found no intragroup significant difference regarding TG. However, there was a significant increase in the average values of TG in the control group and values went beyond the reference value for children (less than or equal to 100 mg/dl), as presented at Table 1.

The control group also presented increased values of TC throughout the study, reaching the limit that is considered as normal for a healthy child (170 to 199 mg/dl). On the other hand, in the experimental group, the TC values continued within the optimum standard (<170 mg/dL) with a slight increase. Children at the control group had a significant decrease of HDL-C values over time (*p*=0.04), reaching an average value inferior to the average shown by the experimental group, which presented an increase of HDL-C values over time. The LDL-C levels reached the optimum values in both groups over time (<110 mg/dl), as presented at Table 1.

The results showed that there was a significant decrease (*p*=0.01) in the HbA1c values in the experimental group (−0.16 ± 0.15). The HDL posttreatment values decreased in the control group and increased in the experimental group, while the LDL values increased in the control group and decreased in the experimental group, as presented in Table 2.

Participants in both groups showed statistically significant increases in weight and height throughout the study (Table 3). In the comparison by groups we did not identify statistically significant differences, at the end of the study, between the experimental and control groups (Table 4).

## 4. Discussion

To the best of our knowledge and based on the current literature available, this is the first randomized clinical trial that examined the effects of cashew nut seed flour in children with moderate malnutrition.

From previous studies it is known that cashew nut flour can exert positive effects on FPG, HbA1c, TG, TC, HDL-C, and LDL-C, directly and indirectly, based on its composition for the lipid metabolism (48.35%), protein (21.76%), starch (17.30%), and total sugar (8.23%) [17].

In this study, we have determined, through both intragroup (experimental) and intergroup analysis (versus experimental group), that children with moderate malnutrition using cashew nut flour showed an improvement (decrease) in glycated hemoglobin value.

Investigations confirm that HbA1c tests are being requested more and more, and so their acceptance as an evidence-based practice has increased. The use of HbA1c parameters was validated in clinical trials with regard to its importance in the evaluation of glycemic control in patients with chronic diseases or complications, as well as its use as a screening test or diagnostic test for diabetes mellitus in children, replacing the fasting plasma glucose and the oral glucose tolerance test (OGTT) [34, 35]. The HbA1c relates to a group of substances consisting of reactions between the hemoglobin A (HbA) and certain sugars. The HbA is the most important and natural form of hemoglobin, while the hemoglobin alpha 1 (HBA1) is a series of glycated variants resulting from attachment of various carbohydrates to N terminal valine of hemoglobin. HbA1c represents the attachment of glucose to N terminal amino acid valine of the beta chain of hemoglobin, leading to an increased negative charge [36].

On the other hand, participants in the control group have shown elevated blood glucose and HDL-C. What may result from this group's weight gain, however, cannot be credited to their diet since eating habits variables were not evaluated during the follow-up in this research.

Red cells have an average lifespan of 120 days. The measurement of the amount of glucose attached to hemoglobin provides a medium glycemic control evaluation within 90 to 120 days before the exam, which makes this parameter an important indicator of the amount of sugar in the body [14]. Studies have shown that the HbA1c represents the overall weighted average of daily blood glucose levels (including fasting and postprandial levels) over the last two to three months [37, 38].

Clinical trials show that the glycemic variability determines the range of variation of glycemic levels at different times throughout the day. In malnutrition, these levels constitute an isolated risk factor independent of mean blood glucose levels, regarding the potential risk for disorders such as cardiovascular diseases [15, 39, 40]. A research has shown that weight gain and height in children do not appear to be significantly altered by glycemic control [41]. The linear growth is mediated by the growth hormone (GH) and by the IGF1 growth factor, which become abnormal during periods of malnutrition.

The ideal concentration of HbA1c in children has not been determined yet, unlike the values for adults. With the increasing prevalence of diseases related to nutrition, especially malnutrition, obesity, and diabetes mellitus in children, the evaluation of HbA1c concentration has become an important parameter in controlling these diseases [37]. Thus, scientists have dedicated themselves to getting more reliable concentrations of HbA1c parameters for children.

A Brazilian study held at the end of the 90 s evaluated two groups of malnourished children, divided into appropriate and inappropriate HbA1c controls. The authors identified significant differences between the groups: height was normal in the group with adequate control, while in the group with inadequate control, a chronological age of more than five years and a longer duration of nutritional damage were detected [42].

These results differ from the present investigation, since the changes in HbA1c occurred due to the experiment, which strengthens the inference of a possible protective effect of the regular use of cashew nut flour.

The glycemic assessment is key to malnourished children care. Among the clinical and metabolic complications of malnutrition, the most relevant disorders include hypoglycemia and hyperglycemia, hypothermia, dehydration, and diarrhea [16].

There is other evidence that changes of blood glucose levels during malnutrition occur due to an increased glucose production from other pathways, reducing insulin synthesis, stimulating the production of glucagon, and increasing circulating epinephrine levels [43].

Since glycogen is rapidly consumed, the body starts the production of glucose through glycerol and free amino acid coming from fatty acids, thus increasing gluconeogenesis [37]. During malnutrition, with increased glucose production by these alternative routes and the increasing reduction in the macronutrient stocks, it becomes difficult to adequately maintain glucose levels, providing a hypoglycemic condition when there is no efficient nutritional supplementation [38].

In our investigation, we suggest that the reduction of HbA1c in moderately malnourished children of the experimental group was related to the use of cashew nut flour, since these children did not have conditions such as hemolytic anemia, hemoglobinopathies, and hypothyroidism, that promote decreases in the level of HbA1c due to the decrease in the number of erythrocytes, hemoglobin, and hematocrit. There are publications that point to the anti-inflammatory and prebiotic effect of cashews [44, 45].

Children who received supplementation with cashew nut flour presented an increase in the serum concentrations of HDL-C and a decrease in the concentrations of LDL-C. These results are consistent with studies which found that an improvement in serum lipids is favorable for enhancing the survival of malnourished children [46].

From a physiological and clinical point of view, serum lipids are constituents of cell membranes increasing its rigidity [46]. HDL-C belongs to a class of lipoproteins produced by the intestine and liver, consisting of phospholipids and apolipoproteins. It has an important function that relates to the transport of cholesterol from peripheral tissues to the liver, therefore reducing the amount of cholesterol in the blood or in cells and reducing the likelihood of diseases associated with hypercholesterolemia [47].

Nutritional deficiencies cause a mechanism in which HDL-C receives part of both LDL-C and apoprotein, contributing to the return of LDL-C to the liver. This allows LDL-C and HDL-C to combine and maintain the balance of cholesterol from cells, where LDL-C carries cholesterol to arteries and HDL-C removes it [45–47].

Therefore, the increase in HDL-C protects the organism against atherosclerosis, since it removes cholesterol from the blood vessels, transporting it to the liver where it is removed from the body. This may explain our findings, since the total cholesterol did not increase in the postexperiment group, suggesting a possible cardiovascular protection in the experimental group.

A study showed that malnutrition and obesity are not the only consequences of nutritional problems, but there are also cardiovascular diseases [48]. A research points out that hypertension is associated with malnutrition, mainly in girls [49, 50].

The product used in our intervention has functional nutritional characteristics that are biologically relevant for health. The cashew nut seed has an excellent nutritional profile, whose composition contains fatty acids (mainly oleic and linoleic acids), phytosterols with 100 to 200 mg of *ß*-sitosterol, high vitamin *E* content, selenium, and insoluble dietary fiber. A study shows that the consumption of these phytochemicals nutrients is directly associated with a reduced risk of cardiovascular diseases and some types of cancer such as colon, rectum, esophagus, prostate, and stomach [47].

Effects of phytosterols on serum lipid profile were demonstrated in 41 studies submitted to a meta-analysis that evaluated the effectiveness of their use in reducing LDL-C. The authors concluded that the ingestion of phytosterols (about 2 g/day) reduced LDL-C by 10%, and when the consumption is associated with a diet low in saturated fat and cholesterol, this reduction can reach up to 20% [51].

Furthermore, another study also highlights the presence of glutamine in cashew nuts [52]. Glutamine is an essential amino acid for the catabolic individuals, such as malnourished, burned, or postoperative patients. The importance of this amino acid in the presence of such health conditions is attributed to its contribution to the synthesis of nucleotides, being a substrate for hepatic gluconeogenesis and an important energy source for the cells of the gastrointestinal epithelium, lymphocytes, fibroblasts, and reticulocytes [53].

Therefore, researchers have verified in laboratory that supplementation with glutamine in immunocompromised mice undergoing bowel resection or malnourished mice improved the immune response and stimulated the intestinal mucosa function [53].

A study in humans confirmed the efficacy of glutamine in the feed for increasing the survival of malnourished and critically ill patients in intensive care units over six months, receiving parenteral nutrition supplemented with 2.5% glutamine [54]. Therefore, the consumption of cashew nut flour contributes to the achievement of essential amino acid requirements, assisting patients in the recovery of health.

There are no studies that show associations or correlations between the use of cashew nut flour and possible biochemical changes in malnourished children. Thus, our positive research findings in relation to HbA1c, HDL-C, and LDL-C parameters should be assessed with caution, considering some existing limitations in the study.

We are not sure that the adherence to the use of the cashew nut flour remained high (≥75%) by the end of the intervention. Although children have been observed monthly by the researchers, it is not possible to guarantee that this use has been regular.

Moreover, in the first month of intervention, two children presented intestinal discomfort due to the use of the cashew nut flour combined with certain types of foods, with a high fat content. Thus, it was necessary to readjust the guidelines for the intake of the flour along with other food groups, which required new strategies to ensure adherence to the product intake.

Based on our findings, we believe that it is important to prolong the exposure to the dietary intervention and to increase the rigor in monitoring compliance in future research on this topic. Thus, further research could lead to more robust inferences about the effects of cashew nut flour on the biochemical parameters as a therapeutic tool for moderately malnourished children. In new studies, it is recommended for the nutritional assessment of children that biochemical and anthropometric markers be added, since, in primary healthcare services, the anthropometric method is more accessible and may be necessary in clinical practice.

Furthermore, it is essential to broaden the research on the effects of this product in populations with other diseases or chronic health conditions.

## 5. Conclusions

Our study showed that a monthly nutritional intervention consisting in the use of cashew nut seed flour, during a 24-week period, had positive effects on glycated hemoglobin, HDL-C, and LDL-C parameters in moderately malnourished children.

## Figures and Tables

**Table 1:** Mean values and standard deviations of biochemical parameters in moderately malnourished children before and after 24 weeks of intervention (*n* = 30), Imperatriz, Brazil, 2017.

	Group control			Experimental group		
	Before	After	*p* value	Before	After	*p* value
Glycemia	72.33 ± 10.08	78.53 ± 12.82	**0.02 ** ^*∗*^	78.00 ± 9.52	78.33 ± 17.15	**0.95 ** ^*∗∗*^
HbA1c^*∗∗∗*^	5.31 ± 0.26	5.28 ± 0.49	**0.82 ** ^*∗∗*^	5.40 ± 0.19	5.24 ± 0.22	**<0.01 ** ^*∗*^
Triglycerides	89.53 ± 33.51	101.20 ± 59.59	**0.47 ** ^*∗∗*^	71.27 ± 32.49	86.53 ± 37.34	**0.21 ** ^*∗*^
Total cholesterol	167.53 ± 20.49	170.93 ± 34.17	**0.58 ** ^*∗*^	160.20 ± 27.51	161.93 ± 23.49	**0.77 ** ^*∗∗*^
HDL	50.60 ± 8.26	43.20 ± 11.34	**0.04 ** ^*∗*^	46.93 ± 12.99	47.07 ± 12.65	**0.96 ** ^*∗∗*^
LDL	99.13 ± 19.02	99.80 ± 33.11	**0.94 ** ^*∗*^	92.80 ± 29.33	97.53 ± 20.58	**0.59 ** ^*∗∗*^

^*∗*^Student's *t*-test for dependent samples (paired groups). ^*∗∗*^Wilcoxon *t*-test. ^*∗∗∗*^Glycated hemoglobin. Source: survey data, 2017.

**Table 2:** Mean values and standard deviations for the differences (before minus after) in the biochemical parameters in moderately malnourished children, after 24 weeks of intervention (*n* = 30), Imperatriz, Brazil, 2017.

	Group control (*N* = 15)	Experimental group (*N* = 15)	*p* value^*∗*^
**Glycemia**	6.20 ± 9.20	20.04 ± 0.33	0.31^*∗*^
**HbA1c ** ^*∗∗∗*^	−0.02 ± 0.45	−0.16 ± 0.15	0.01^*∗∗*^
**Triglycerides**	11.67 ± 61.89	15.27 ± 45.53	0.31^*∗∗*^
**Total cholesterol**	23.44 ± 3.40	22.31 ± 1.73	0.84^*∗*^
**HDL**	−7.40 ± 12.59	10.33 ± 0.13	0.08^*∗∗*^
**LDL**	14.28 ± 7.86	−1.26 ± 22.30	0.33^*∗∗*^

^*∗*^Student's *t*-test for independent samples. ^*∗∗*^Wilcoxon–Mann–Whitney test. ^*∗∗∗*^Glycated hemoglobin. Source: survey data, 2017.

**Table 3:** Mean values ± standard deviations of anthropometric measurements of underweight or moderately malnourished children, before and after treatment, according to the group (experimental and control), Imperatriz, Brazil, *n* = 30, 2017.

	Control group			Experiment group		
	Before	After	*p* ^*∗*^ value	Before	After	*p* ^*∗*^ value
Height (cm)	92.33 ± 13.53	95.80 ± 13.64	<0.001	100.93 ± 12.19	104.13 ± 10.95	**<0.001**
Weight (kg)	13.55 ± 3.46	14.11 ± 3.17	<0.001	15.03 ± 3.78	15.82 ± 3.98	**<0.001**
BMI^*∗∗*^	15.80 ± 1.55	15.41 ± 1.97	0.13	14.63 ± 1.60	14.44 ± 1.39	**0.38**

^*∗*^Student's *t*-test for dependent samples (paired groups). ^*∗∗*^Body mass index.

**Table 4:** Mean values ± standard deviations for differences (after/before) in anthropometric measurements of underweight or malnourished children according to the group (experimental and control), Imperatriz, Brazil, *n* = 30, 2017.

	Control group	Experimental group	*p* ^*∗*^ value
Height (cm)	3.46 ± 2.09	3.20 ± 2.11	0.73
Weight (kg)	0.55 ± 0.68	0.79 ± 0.59	0.33
BMI^*∗∗*^	−0.38 ± 0.92	−0.18 ± 0.80	0.53

^*∗*^Student's *t*-test for dependent samples (paired groups). ^*∗∗*^Body mass index.

## Data Availability

The data used to support the findings of this study are available from the corresponding author upon request.
